# Household Hints and Recipes

**Published:** 1873-10

**Authors:** 


### Household Hints & Recipes.

A Useful Soap.—The following is commended by those who have tried it for scrubbing and cleansing painted floors, washing dishes, and other household purposes: Take two pounds of white olive soap and shave it in thin slices; add two ounces of borax and two quarts of cold water; stir all together in a stone or earthen jar, and let it be set upon the back of the stove until the mass is dissolved. A very little heat is required, as the liquid need not simmer. When thoroughly mixed and cooled, it becomes of the consistence of a thick jelly, and a piece the size of a cubic inch will make a lather for a gallon of water.

---

To Clean Gold Chains.—Put the chain in a small glass bottle with warm water, a little tooth powder, and some soap. Cork the bottle and shake it for a minute violently. The friction against the glass polishes the gold, and the soap and chalk extract every particle of grease and dirt from the interstices of a chain of the most intricate pattern; rinse it in clear cold water, wipe with a towel, and the polish will surprise you.

---

Wax for Floors.—Heat 5 ounces of pearl-ash, 25 ounces of wax, and 20 ounces of water together, stirring it frequently until a thick mass results, from which water does not separate on standing a short time. Then add from a pint to a pint and a half of boiling water, with constant stirring. This may be colored if desired. It may be applied with a woolen rag, and thoroughly rubbed in.

---

Blackberry Wine.—An exchange gives the following as "Senator Wilson's way" of making blackberry wine :—

Measure your berries and bruise them ; to every gallon adding one quart of boiling water. Let the mixture stand twenty-four hours, stirring occasionally; then strain off the liquor into a cask, to every gallon adding two pounds of sugar; cork tight, and let it stand to the following October, and the wine is ready for use without further straining or boiling.

And this is said to be "Senator Henkle's way:" Take 100 quarts of blackberries, crush them and press out the juice. Then dissolve 110 pounds of white sugar in 20 gallons cold water. Measure the syrup; add the juice, and as much more water as will be required to make 40 gallons in all. If you want to make a smaller quantity, preserve the above proportions. After putting it in the cask (one that has recently had whiskey is preferred), set it in the cellar or other cool place with the bung open to the air until Christmas. Then stop tightly or bottle it.

---

Blackberry Cordial.—To two quarts of juice add one pound of white sugar; half ounce nutmeg; half ounce cloves pulverized. Boil all together for a short time, and when cold add a pint of brandy.

This syrup is said to be almost a specific for summer complaint or diarrhœa. From a teaspoonful to a wine-glass is to be taken, according to the age of the patient, until relieved.

---

Tomato Honey.—To each pound of tomatoes allow the grated peel of a lemon and six fresh peach leaves. Boil them slowly till they are all to pieces, then squeeze them through a bag. To each pound of liquid allow a pound of sugar and the juice of one lemon.—Boil them together half an hour, or till they become a thick jelly. Then put them into glasses, and lay double tissue paper over the top. It will be scarcely distinguished from real honey.

---

Remedy for a Chafed Skin.—Dr. Hudson has recommended, in the *Pacific Med. and Surg. Journal*, a powder which, he says, is a most efficient and elegant preparation for the chafed skins of infants; it is composed of

| | |
|---|---|
| Soap stone, powdered . . . | 2 parts. |
| Calomel . . . . . . | 1 part. |

---

Lotion of Acetic Acid for Baldness.—The following lotion is said to be superior for a shampooing liquid, for removing dandruff, and useful and pleasant application for baldness. It is, of course, moderately stimulating, and in those cases in which the hair-follicles are not destroyed, but have become merely inactive, we should think it might prove both efficacious and agreeable :

| | |
|---|---|
| Take of acetic acid . . . | 1 drachm. |
| Cologne water . . . . | 1 ounce. |
| Water, to make in all . . | 6 ounces. |

Mix.

Hygienic Use of Tea.—In a paper by Dr. Adam Smith, read before the London Society of Arts, the use of tea was recommended in the following cases:—After a full meal, when the system is oppressed; for the corpulent and the old; for hot climates, and especially for those who, living there, eat freely, or drink milk or alcohol; in cases of suspended animation; for soldiers who, in time of peace, take too much food in relation to the waste proceeding in the body; for soldiers and others marching in hot climates, for then, by promoting evaporation and cooling the body, it prevents in a degree the effects of too much food, as of too great heat.

---

Calomel for Piles.—Calomel applied once or twice a day to tumid and tender piles rarely fails to cure them in a few days.

---

A Good Cheap Cologne.—The *Druggists' Circular* gives this recipe:

| | |
|---|---|
| Oil of bergamot, | |
| " orange, | |
| " rosemary, of each | 1 fl. drachm. |
| Orange flower water, | |
| Alcohol, of each . . . | 1 pint. |
| Cardamon seeds . . | 1 drachm. |

Mix, digest, and distil over one pint.

---

A Safe Hair-Dressing.—The following is from the *Year Book of Pharmacy* for 1872 :—

| | |
|---|---|
| Oil of cocoanut . . . | 12 ounces. |
| Castor oil . . . . . | 3 pounds. |

Melt the cocoanut oil and add then the castor oil; agitate until they are thoroughly mixed, and add

| | |
|---|---|
| Strong alcohol . . . . . | 4 pints. |

---

French Putty.—7 lbs. linseed oil and 4 lbs. brown umber are boiled for two hours, and 62 grammes wax stirred in. After removal from the fire, 5½ lbs. fine chalk and 11 lbs. white lead are added and thoroughly incorporated. This putty is said to be very hard and permanent.

---

Whitewashing.—A farmers' journal gives the following receipt for making a superior whitewash:—Take a clean, water-tight barrel or other suitable cask, and put in it half a bushel of lime. Slake it by pouring water over it, boiling hot, and in sufficient quantity to cover it five inches deep, and stir it briskly till thoroughly slaked. When the slaking has been effected, dissolve it in water, and add a solution of two pounds of sulphate of zinc, and one of common salt. As it is often desirable to vary the monotony by introducing a variety of colors upon the premises, it may be important to know that a beautiful cream color may be communicated to the above wash by adding to it three pounds of yellow ochre; or a good pearl or lead color by the addition of lamp, vine, or ivory black. For fawn color, add four pounds umber—Turkish or American (the latter is the cheapest), one pound Indian red, and one pound lampblack. For common stone color, add four pounds raw umber, and two pounds lamp-black. The value of the sulphate of zinc is that it makes the wash harden better after it is put on.—Some also put in a half pound of common alum; others substitute the alum for the zinc. There is no mode of making farm buildings attractive so cheaply as with whitewash.

---

Oiling Harness.—A correspondent of the *Prairie Farmer* gives the following good, plain, practical directions for oiling harness, and although the mode has been long familiar to many of our readers, it may be useful to many others:—

Harness should never be used over six months without oiling. I take my harness apart, or unbuckle it as much as convenient, and put it into the soap-suds of the last washing, giving it a good soaking; then take it out upon a bench of the proper height for convenience, and remove all the dirt and gummy substance with the assistance of a stiff brush and the soap-suds. I then find it just the time to make necessary repairs. By this time the water has dried from the surface, and I then apply the oil and a little lampblack mixed with a common paint-brush, and dry in the shade; if frozen, all the better. When dry, wipe off with a cloth, and put together.

It is my opinion, founded upon a forty years' experience and close observation, that harness treated in this manner will last twice as long as it will with proper oiling and care. The more leather is neglected, the darker it will become, inwardly, and the darker the less life remains, and the less good will oil do when applied to recuperate it.

Ammoniacal fumes arising in the stable have a strong tendency to eat up the oil in the harness; hence the desirability of storing harness out of the reach of this influence.

Gold Varnish for Picture Frames.—Turmeric, gamboge, of each one drachm; spirits of turpentine, two pints; shellac, sandarach, of each five ounces; dragon's blood, seven drachms; thin mastic varnish, eight ounces. Digest with occasional agitation, for fourteen days, in a warm place; then set aside to fine, and pour off the clear liquid. This is to be applied on the frames previously silvered.

---

Washing Compound.—The use of soda for washing linen is very injurious to the tissue, and imparts to it a yellow color. In Germany and Belgium the following mixture is now extensively and beneficially used: 2 lbs. of soap are dissolved in about 5 gallons of water as hot as the hand can bear it; then next is added to this fluid three large-sized table-spoonfuls of liquid of ammonia and one spoonful of best oil of turpentine. These fluids are incorporated rapidly by means of beating them together with a small birch broom. The linen is then soaked in this liquid for three hours, care being taken to cover the washing-tub by a closely-fitting wooden cover. By this means the linen is thoroughly cleaned, saving much rubbing, time, and fuel. Ammonia does not affect the linen or woolen goods, and is largely used as a washing liquor in the North of England.

---

Whitening Smoked Walls.—A method of cleaning and whitening smoked walls consists, in the first place, of rubbing off all black loose dirt upon them by means of a broom, and then washing them down with a strong soda lye, which is to be afterward removed by means of water to which a little hydrochloric acid has been added. When the walls are dry, a thin coating of lime with the addition of a solution of alum is to be applied. After this has become perfectly dry the walls are to be kalsomined, or coated with a solution of glue and chalk.—*Mining and Scientific Press.*

---

Polish for Patent Leather.—The following is given by the London *Chemist and Druggist*:—

Whites of two eggs.

One tablespoonful of spirits of wine.

Two large lumps of sugar.

Finely-powdered ivory-black, as much as may be sufficient to produce the necessary blackness and consistence. To be laid on with a soft sponge lightly, and afterwards gently rubbed with a soft cloth.

To keep Gum-Arabic from Moulding.—Solutions of gum-arabic soon mould and sour, and finally lose their adhesive property. It is said that sulphate of quinine will prevent this, while it imparts no bad odor of its own. The addition of a solution of a few crystals of this salt to gum-arabic will prevent the formation of mould quite as effectually as carbolic acid, and by analogy it is safe to suppose that the same salt could be used in glue.

---

Adhesive Paste for Bottle Labels.—Four parts by weight of glue are allowed to soften in 15 parts of cold water for some hours, and then moderately heated till the solution becomes quite clear. Sixty-five parts of boiling water are now added with stirring. In another vessel 30 parts of starch paste are stirred up with 20 parts cold water, so that a thin milky fluid is obtained without lumps. Into this the boiling glue solution is poured, with constant stirring, and the whole is kept at the boiling temperature. After cooling, 10 drops of carbolic acid are added to the paste. This paste is of extraordinary adhesive power, and if preserved in closed bottles to prevent evaporation of the water, will keep good for years.

---

Gold Powder.—Gold powder for gilding may be prepared by putting into an earthen mortar some gold leaf, with a little honey or thick gum-water, and grinding the mixture till the gold is reduced to extremely minute particles. When this is done, a little warm water will wash out the honey or gum, leaving the gold behind in a pulverulent state. Another way is to dissolve pure gold, or the leaf, in nitro-muriatic acid, and then to precipitate it by a piece of copper, or by a solution of sulphate of iron. The precipitate (if by copper) must be digested in distilled vinegar and then washed (by pouring water over it repeatedly) and dried. This precipitate will be in the form of a very fine powder. It works better and is more easily burnished than gold-leaf ground in honey as above.

---

How to Swallow a Pill.—The *Chicago Medical Times* is responsible for the following: "Put the pills under the tongue and behind the teeth, and let the patient immediately take a large swallow of water, and he will neither feel the pill nor taste it. In fact, he cannot tell where it has gone, and I have seen them look about the floor to see if they had not dropped it."

